# Youth Exposure to Alcohol Advertising on Television — 25 Markets, United States, 2010

**Published:** 2013-11-08

**Authors:** David H. Jernigan, Craig S. Ross, Joshua Ostroff, Lela R. McKnight-Eily, Robert D. Brewer

**Affiliations:** Johns Hopkins Univ, Baltimore, MD; Virtual Media Resources, Natick, MA; Div of Population Health, National Center for Chronic Disease Prevention and Health Promotion, CDC

Excessive alcohol consumption accounted for an estimated 4,700 deaths and 280,000 years of potential life lost among youths aged <21 years each year during 2001–2005 (1). Exposure to alcohol marketing increases the likelihood to varying degrees that youths will initiate drinking and drink at higher levels (2). By 2003, the alcohol industry voluntarily agreed not to advertise on television programs where >30% of the audience is reasonably expected to be aged <21 years. However, the National Research Council/Institute of Medicine (NRC/IOM) proposed in 2003 that “the industry standard should move toward a 15% threshold for television advertising” (3). Because local media markets might have different age distributions, the Center on Alcohol Marketing and Youth, Johns Hopkins Bloomberg School of Public Health, evaluated the proportion of advertisements that appeared on television programs in 25 local television markets* and resulting youth exposure that exceeded the industry standard (i.e., >30% aged 2–20 years) or the proposed NRC/IOM standard (i.e., >15% aged 12–20 years). Among national television programs with alcohol advertising, placements were assessed for the 10 programs with the largest number of youth viewers within each of four program categories: network sports, network nonsports, cable sports, and cable nonsports (40 total). Of the 196,494 alcohol advertisements that aired on television programs with the largest number of youth viewers in these local markets, placement of 23.7% exceeded the industry threshold and 35.4% exceeded the NRC/IOM threshold. These results indicate that the alcohol industry’s self-regulation of its advertising could be improved, and youth exposure to alcohol advertising could be further reduced by adopting and complying with the NRC/IOM standard. In addition, continued public health surveillance would allow for sustained assessment of youth exposure to alcohol advertising and inform future interventions.

Nielsen Station Index Local People Meter Market Survey† data for 2010 were used to assess exposure to alcohol advertisements placed on nationally telecast programs among a sample of households in 25 local media markets, as well as the demographic characteristics of program viewers aged ≥2 years in these markets (approximately 98.9% of all U.S. households have televisions) (4). In 2010, these 25 media markets were among the largest in the United States and accounted for 50.3% of the total U.S. population aged 12–20 years living in homes with televisions (5).

Advertising exposure was analyzed first using the current voluntary industry standard, which calls for no alcohol advertising during programs for which persons aged 2–20 years composed >30% of the expected audience. Exposure also was analyzed using the NRC/IOM proposed standard that called on industry to move toward a 15% threshold for television advertising using persons aged ≥12 years as the denominator.§ Alcohol use usually begins in early adolescence; federal surveys begin measuring youth drinking at age 12 years, and age 21 years is the minimum legal age for the purchase of alcohol in all 50 states. The local population was used as the denominator to account for differences in the age distribution of local media markets.

Among nationally televised programs with alcohol advertising, exposure to this advertising was evaluated for the 10 programs with the largest number of youth viewers in each of four program categories: cable sports, cable nonsports, broadcast network sports, and broadcast network nonsports (i.e., 40 programs in total) in each of the 25 television media markets. Nationally, these programs represented 29% of all youth exposure to alcohol advertising on broadcast network nonsports, 20% on broadcast network sports, 20% on cable sports, and 14% on cable nonsports. The total number of gross impressions,¶ an indicator used by the advertising industry to measure advertising exposure, was calculated by summing the placement-specific number of viewers of different ages across all advertising placements for a particular market. A total of 196,494 alcohol product advertisements aired on the 40 programs that were assessed across the 25 markets, or approximately 7,860 advertisements per market; however, not all advertisements appeared in all markets.

Of the 196,494 total alcohol advertisements, 46,493 (23.7%) were placed during programs for which >30% of the audience was aged 2–20 years (range: 31.5% in Houston, Texas, to 16.3% in Washington, DC); and 69,622 (35.4%) were placed during programs that exceeded the 15% threshold (range: 45.2% in Chicago, Illinois, to 25.9% in Portland, Oregon) (Table 1).** Of the 797,571,000 total alcohol advertising impressions among youths aged 12–20 years that resulted from these advertisements, 33.3% were from advertisements that were placed in programs exceeding the 30% threshold (range: 45.4% in Orlando-Daytona Beach-Melbourne, Florida, to 25.2% in Washington, DC); and 54.4% were from advertisements on programs that exceeded the 15% threshold (range: 65.3% in New York, New York, to 42.0% in Boston, Massachusetts) (Table 2).††

## Editorial Note

The findings in this report indicate that in 25 of the largest television markets in the United States, almost one quarter of the alcohol advertisements airing on this sample of national television programs popular with youths had local underage audiences >30%, exceeding the alcohol industry’s voluntary 2003 self-regulatory codes, and more than one third aired during programs that exceeded the NRC/IOM recommended threshold of 15% youth audience composition. Although the total number of advertising occurrences was consistent in each of the 25 markets, youth exposure to alcohol advertising exceeding the 30% standard varied across these markets. If the advertising exceeding the industry threshold of 30% were eliminated and not replaced, total youth exposure to alcohol advertising on these programs would drop by one third. If alcohol companies were to eliminate and not replace advertisements above the NRC/IOM recommended limit of 15%, total youth exposure to alcohol advertising on these programs would drop by an estimated 54%.

From 2001 to 2009, youth exposure to alcohol advertising on television in the United States increased by 71% (6). This is largely attributable to increased alcohol advertising on cable television programs, particularly by distilled spirits companies (6). The increase in spirits advertising on cable television also coincides with an observed increase in consumption of spirits by high school students, particularly among those who binge drink (i.e., consume ≥5 drinks on an occasion for males and ≥4 drinks on an occasion for females) (7).

The findings in this report are subject to at least three limitations. First, the 25 media markets might not be broadly representative of the United States, although they are likely to represent major metropolitan areas. Second, this study is limited by its focus on national television advertisements delivered through broadcast or cable delivery and does not assess potential exposure to alcohol advertising on streamed television programming delivered through the Internet. Finally, youth exposure to alcohol advertising was assessed using a sample of 40 television programs with alcohol advertising that were also known to have the largest youth audiences in each of the four program categories; thus, the findings are unlikely to be representative of youth exposure to alcohol advertising on all television programs. Nonetheless, these findings are likely to be representative of alcohol advertising placed on national television programs that are popular with youths in major metropolitan areas.

The results of this evaluation suggest that the alcohol industry has not consistently met its 2003 self-regulatory standards to avoid airing alcohol advertising during programs where >30% of the audience is underage, and that industry marketing codes would benefit from the use of local as well as national data on the age distribution and television use of viewing audiences. In 1999, the Federal Trade Commission also recommended that the industry develop “no-buy” lists barring alcohol advertising on television programs and in other media that are likely to have disproportionately large underage audiences (8). Strategies recommended by the U.S. Community Preventive Services Task Force to reduce excessive alcohol use include increasing alcohol excise taxes and regulating alcohol outlet density (9). Continued public health surveillance of youth exposure to alcohol advertising allows for the ongoing monitoring of compliance with marketing standards, and can help inform the planning, implementation, and evaluation of interventions to further reduce youth exposure to alcohol marketing.

What is already known on this topic?

Youth exposure to alcohol advertising is associated with the initiation of alcohol use and higher levels of consumption among youths who drink. The alcohol industry uses voluntary advertising codes based on youth audience composition to guide the placement of alcohol advertising, but compliance with these voluntary codes has not been evaluated at the local level.

What is added by this report?

In 25 of the largest television markets in the United States, approximately one in four alcohol advertisements on a sample of 40 national TV programs popular with youths had underage audiences >30%, exceeding the alcohol industry’s voluntary 2003 self-regulatory codes.

What are the implications for public health practice?

If the alcohol advertising on the popular national television programs in the 25 largest television markets where >30% of the local audience was underage were eliminated and not replaced, total youth exposure to alcohol advertising on these programs could drop by as much as one third. Continued public health surveillance of youth exposure to alcohol advertising will allow for the ongoing assessment of compliance with marketing codes and can help inform the planning, implementation, and evaluation of interventions to further reduce youth exposure to alcohol marketing.

## Figures and Tables

**TABLE 1:** Number and percentage of television alcohol advertisements that aired locally during programming viewed by greater than recommended percentages of underage youths,* by audience composition and television market — United States, 2010

Market	Local market population aged 12–20 yrs (%)	Total no. of advertising occurrences	Advertisements in programming that exceeded youth audience composition threshold			

			>30% audience		>15% audience	
	
			No.	(%)	No.	(%)
**Top five markets by proportion >30%**						
Houston, Texas	(13.7)	7,862	2,476	(31.5)	3,256	(41.4)
Los Angeles, California	(13.8)	7,869	2,364	(30.0)	3,509	(44.6)
Dallas-Ft. Worth, Texas	(13.2)	7,862	2,334	(29.7)	3,055	(38.9)
Atlanta, Georgia	(12.9)	7,859	2,169	(27.6)	3,103	(39.5)
Chicago, Illinois	(13.3)	7,862	2,160	(27.5)	3,550	(45.2)
**Bottom five markets by proportion >30%**						
Seattle-Tacoma, Washington	(11.8)	7,869	1,469	(18.7)	2,711	(34.5)
San Francisco-Oakland-San Jose, California	(11.3)	7,869	1,447	(18.4)	2,365	(30.1)
Boston (Manchester), Massachusetts	(12.0)	7,844	1,367	(17.4)	2,124	(27.1)
Sacramento-Stockton-Modesto, California	(13.6)	7,869	1,339	(17.0)	2,337	(29.7)
Washington, DC (Hagerstown)	(12.1)	7,859	1,284	(16.3)	2,062	(26.2)
**Total (all markets)**	**—**	**196,494**	**46,493**	**(23.7)**	**69,622**	**(35.4)**

**Source:** The Nielsen Company, New York, New York.

* Aged 12–20 years. The alcohol industry voluntarily agreed not to advertise on television programs where >30% of the audience is reasonably expected to be aged <21 years, here assessed as viewers ages 2–20 years. The National Research Council/Institute of Medicine proposed that “the industry standard should move toward a 15% threshold for television advertising,” assessed here for programming for which >15% of all viewers aged ≥12 years were aged 12–20 years.

**TABLE 2:** Proportion of television alcohol advertising exposures to underage youths* that exceeded voluntary and proposed thresholds† for underage audience composition, by market — United States, 2010

Local markets	Local market population aged 12–20 yrs (%)	Total no. of youth advertising exposures (000s)	Youth television advertising exposures exceeding audience composition thresholds (%)	

			>30% audience	>15% audience
**Top five markets by proportion >30%**				
Orlando-Daytona Beach-Melbourne, Florida	(11.6)	24,078	(45.4)	(59.6)
Houston, Texas	(13.7)	32,683	(43.0)	(61.6)
Pittsburgh, Pennsylvania	(11.9)	13,319	(40.9)	(59.1)
Tampa-St. Petersburg (Sarasota), Florida	(11.0)	24,326	(40.8)	(57.9)
Detroit. Michigan	(13.3)	25,749	(39.0)	(63.5)
**Bottom five markets by proportion >30%**				
Charlotte, North Carolina	(12.3)	15,833	(29.4)	(56.9)
San Francisco-Oakland-San Jose, California	(11.3)	22,484	(29.3)	(47.1)
Boston (Manchester), Massachusetts	(12.0)	28,858	(26.5)	(42.0)
Sacramento-Stockton-Modesto, California	(13.6)	17,567	(25.6)	(48.4)
Washington, DC (Hagerstown)	(12.1)	27,022	(25.2)	(43.7)
**Total impressions**	**—**	**797,571**	**(33.3)**	**(54.4)**

**Source:** The Nielsen Company, New York, New York.

* Aged 12–20 years.

† The alcohol industry voluntarily agreed not to advertise on television programs where >30% of the audience is reasonably expected to be aged <21 years, here assessed as viewers ages 2–20 years. The National Research Council/Institute of Medicine proposed that “the industry standard should move toward a 15% threshold for television advertising,” assessed here for programming for which >15% of all viewers aged ≥12 years were aged 12–20 years.

## Footnotes

* Television media markets studied included Atlanta, Georgia; Baltimore, Maryland; Boston, Massachusetts; Charlotte, North Carolina; Chicago, Illinois; Cleveland, Ohio; Dallas, Texas; Denver, Colorado; Detroit, Michigan; Houston, Texas; Los Angeles, California; Miami, Florida; Minneapolis, Minnesota; New York, New York; Orlando, Florida; Philadelphia, Pennsylvania; Phoenix, Arizona; Pittsburgh, Pennsylvania; Portland, Oregon; Sacramento, California; San Francisco, California; Seattle, Washington; St. Louis, Missouri; Tampa, Florida; and Washington, DC. These 25 media markets represent 25 of the 26 largest television markets by population. Raleigh-Durham, North Carolina, the 25th largest market, was excluded because it did not have Nielsen Local People Meter data at of the time of this analysis.

† Introduced by Nielsen in 2002, Local People Meters measure viewing behavior and viewer demographics and have been phased into the largest television markets over the past decade. In comparison with traditional paper diary methods, or with earlier-generation channel-tuning meters supplemented by paper diaries to obtain demographic viewing estimates, Local People Meters are more precise and are now widely accepted by advertisers, television networks, and television stations as the standard for measuring local viewing in larger markets.

§ The rationale for 30% was to limit advertisements to media in which the legal-age adult audience (aged ≥21 years) was proportional to the legal-age adult population, at that time 70%. This standard has most recently been revised to 28.4% underage (71.6% legal age) based on 2010 census data. However, not all youths are at equal risk for drinking. For example, few youths ages 2–11 years engage in drinking behaviors, and the youngest age at which federal surveys begin measuring drinking behavior is 12 years. Thus, the 15% standard is based on the at-risk population of youths aged 12–20 years, which makes up approximately 15% of the U.S. population aged ≥12 years.

¶ An advertising impression occurs when one person sees an advertisement. If an advertisement is seen by five different people, that counts as five impressions. Gross impressions are the sum of impressions for any given advertising campaign, and include multiple exposures for some or all of the people who are exposed to that campaign.

** Table 1 shows the top and bottom five markets with youth audiences in excess of 30%. Portland was the low market on the 15% standard, but was not in the bottom five for the 30% standard, so it does not appear in the table.

†† Data for all 25 markets available at http://www.camy.org.
